# Management of Adverse Reactions for BCMA-Directed Therapy in Relapsed Multiple Myeloma: A Focused Review

**DOI:** 10.3390/jcm12175539

**Published:** 2023-08-25

**Authors:** Razwana Khanam, Beth Faiman, Saba Batool, Mohammed Musa Najmuddin, Rana Usman, Kiran Kuriakose, Arooj Ahmed, Mohammad Ebad Ur Rehman, Zinath Roksana, Zain Syed, Faiz Anwer, Shahzad Raza

**Affiliations:** 1 Department of Hospital Medicine, Baystate Medical Center, Springfield, MA 01199, USA; 2 Department of Hematology and Medical Oncology, Cleveland Clinic Taussig Cancer Institute, Cleveland, OH 44106, USA; faimanb@ccf.org (B.F.); anwerf@ccf.org (F.A.); razashahzad2@gmail.com (S.R.); 3 Department of Hospital Medicine, Carle Health Methodist Hospital, Peoria, IL 61636, USA; saba.m.batool@gmail.com; 4 The Wright Center for Graduate Medical Education, Scranton, PA 18505, USA; musanajmudinn@gmail.com; 5 University of Tennessee Health Science Center, Memphis, TN 38163, USA; rusman@uthsc.edu; 6 Department of Hospital Medicine, UPMC Mercy Hospital, Pittsburgh, PA 15219, USA; kuriakosek@upmc.edu; 7 Department of Translational Hematology and Oncology, Cleveland Clinic Taussig Cancer Center, Cleveland, OH 44195, USA; aroojumar25@hotmail.com; 8 Department of Medicine, Rawalpindi Medical University, Rawalpindi 46000, Pakistan; ebadrehman.rehman@gmail.com; 9 Sheikh Hasina National Institute of Burn and Plastic Surgery, Dhaka 1217, Bangladesh; zinathzuthy@gmail.com; 10 Department of Biomedical Engineering, Case Western Reserve University, Cleveland, OH 44106, USA; zms39@case.edu

**Keywords:** BCMA, Bispecific antibodies, CAR-T cell therapy, relapsed multiple myeloma, BiTEs, ADCs

## Abstract

Anti-B-cell maturation antigen therapies consisting of bispecific antibodies, antibody–drug conjugates, and chimeric antigen receptor T cells have shown promising results in relapsed refractory multiple myeloma (RRMM). However, the severe side effects include cytokine release syndrome, immune effector cell-associated neurotoxicity syndrome, cytopenia(s), infections, hemophagocytic lymphohistiocytosis, and organ toxicity, which could sometimes be life-threatening. This review focuses on these most common complications post-BCMA therapy. We discussed the risk factors, pathogenesis, clinical features associated with these complications, and how to prevent and treat them. We included four original studies for this focused review. All four agents (idecabtagene vicleucel, ciltacabtagene autoleucel, teclistamab, belantamab mafodotin) have received FDA approval for adult RRMM patients. We went through the FDA access data packages of the approved agents to outline stepwise management of the complications for better patient outcomes.

## 1. Introduction

Multiple myeloma (MM) remains incurable despite the advances made with the discovery of many novel drugs and three- to four-drug combination regimen therapy consisting of alkylators, proteasome inhibitors (PI), immunomodulatory agents (IMiD), corticosteroids, histone deacetylase inhibitors (HDAC inhibitors), and monoclonal antibodies [1]. Treatment failure is seen mainly secondary to resistance to these therapeutic agents, with a death rate of 3.1 per 100,000 population per year, as seen in SEERS 2016–2020 [1,2]. 

B-cell maturation antigen (BCMA) is a member of the tumor necrosis factor (TNF) receptor superfamily, usually found on the surface of normal B-lymphocytes; however, it is overexpressed in pathogenic plasma cells [3]. BCMA has two ligands, a proliferation-inducing ligand (APRIL) and a B-cell activating factor (BAFF), where APRIL has a greater affinity to bind to its receptor when compared to BAFF [4]. These ligands activate the downstream signals, such as nuclear factor kappa-B, rat sarcoma/mitogen-activated protein kinase, and phosphoinositide-3-kinase-protein kinase B/Akt, activating anti-apoptotic protein resulting in cell survival and proliferation [5,6,7]. BCMA can be targeted in three different ways: chimeric antigen receptor T (CAR-T) cell therapy, bispecific antibodies (BsAbs), and antibody-drug conjugates (ADCs). 

In 2017, the efficacy of anti-BCMA CAR-T therapy was first published on relapsed/refractory multiple myeloma (RRMM) patients [8,9]. CAR-Ts, including idecabtagene vicleucel (ABECMA, March 2021) and ciltacabtagene autoleucel (Carvykti, February 2022), are genetically modified autologous T-cells received approval from Food and Drug Administration (FDA) for use in RRMM after four or more prior lines of therapy, including an ImiD, a PI, and an anti-CD38 monoclonal antibody [10,11]. Belantamab mafodotin is the first BCMA/ADC that received accelerated approval by the FDA to be used in adult RRMM; however, it was later withdrawn from the US market in November 2022 as the DREAMM-3 phase 3 trial did not meet its primary endpoint [12]. It is currently used in some circumstances as an expanded access program. On the other hand, teclistamab is the first bispecific antibody to attain accelerated approval by the FDA for adult RRMM who have received at least four prior lines of therapy including a PI, an IMiD, and an anti-CD38 monoclonal antibody [13]. 

To date, the BCMA-targeting agent has shown 70–100% overall responses in heavily treated RRMM [14]. However, patients can encounter life-threatening complications such as cytokine release syndrome (CRS), immune effector cell-associated neurotoxicity syndrome (ICANS), cytopenia(s), infections, hemophagocytic lymphohistocytosis, and other systemic toxicities. In this review, we have discussed common toxicities associated with anti-BCMA therapy and management of these toxicities. 

## 2. Method

We have mainly focused on four studies based on three predetermined criteria: original completed phase I/II clinical trials, BCMA agents that received FDA approval, relapsed refractory multiple myeloma, and age > 18 years [15,16,17,18]. All four agents (idecabtagene vicleucel, ciltacabtagene autoleucel, teclistamab, belantamab mafodotin) have already been approved by the FDA for use in adult RRMM. We have also reviewed these agents’ FDA access data packages and highlighted their recommendations made for treating the complications [19,20,21,22].

## 3. Types of BCMA Agents

As mentioned earlier, BCMA can be targeted in three different ways:

1. Chimeric antigen receptor T (CAR-T) cell therapy
2. Bispecific antibodies (BsAbs), and
3. Antibody-drug conjugates (ADCs)

Chimeric antigen receptor-T cells (CAR-T) are genetically modified T cells that express chimeric antigen receptors (CAR) that could beautologous vs. allogeneic, consisting of three domains: an extracellular (usually from a single-chain variable fragment of an antibody), an intracellular, and a co-stimulatory domain [14]. These cells bind to tumor antigens in a major histocompatibility complex (MHC)-independent manner, resulting in cytotoxic T-cell mediated tumor lysis [23]. To date, the FDA has approved two autologous CAR-T cells for adult RRMM patients who received four or more prior lines of therapy, including an IMiD, a PI, and an anti-CD38 monoclonal antibody [10,11]. The approval of idecabtagene vicleucel (ide-cel) was granted after a single-arm study (KarMMa, NCT03361748) on patients with RRMM; the multicenter multicohort phase II of this study (KarMMa-2) is ongoing (*n* = 31), which showed an overall response rate (ORR) of 87.1% with complete response/stringent complete response (CR/sCR) in 74.2%, very good partial response (VGPR) in 3%, and partial response (PR) in 1% patients after a median follow up (mFU) of 27.5 months [15]. An open-label phase III study (KarMMa-3, NCT03651128) assessed the difference in response in RRMM patients to ide-cel versus standard therapy. These patients previously received two to four regimens, including an IMiD, PI, and daratumumab. The ORR was recorded at 71% (CR in 39%) in ide-cel cohort, and 42% (CR in 5%) in the standard therapy cohort after a mFU of 18.6 months. The median progression-free survival (mPFS) was 13.3 and 4.4 months in the ide-cel and standard-therapy group, respectively [24]. The approval of the second CAR-T therapy ciltacabtagene autoleucel (cilta-cel) for RRMM came after the CARTITUDE-1 trial (NCT03548207), a phase Ib/II study (*n* = 97), in which eligible patients received ≥ 3 prior line of therapy (LOT) or were double refractory to a PI and IMiD. ≥CR was achieved in 78.3% of the cases after an mFU of 33.4 months. The median duration of response (mDOR) was 33.9 months, and the mPFS was 34.9 months [16]. In CARTITUDE-2 (NCT4133636), a phase II study, which is being conducted on three different cohorts, cohort A (*n* = 20) has patients with progressive MM after 1–3 prior LOT (including a PI and IMiD) with no previous exposure to BCMA, cohort B (*n* = 19) has patients with early progressive MM with one prior LOT without any exposure to BCMA, and cohort C (*n* = 20) has patients with progressive MM after exposure to BCMA-targeting agent. The ORR was recorded at 95% (≥CR in 85% and ≥VGPR in 95%), 100% (≥CR in 90% and ≥VGPR in 100%), and 60% (≥CR in 30% and ≥VGPR in 55%) in cohort A, B, and C, respectively [25,26,27]. Recently, a phase 1 clinical trial (NCT03287804) got published, in which dual targeting CAR was constructed to target two myeloma antigens and BCMA. In this study (*n* = 11), patients received a median of 5 prior LOT, with 81.8% being double-refractory and 18.2% penta-refractory. A total of 45.5% responded, with 9% showing VGPR, 27.3% PR, and 9% a minimal response. The mPFS was five months, and the median overall survival (mOS) was 375 days [28].

Bispecific antibodies (BsAbs) are molecules that bind simultaneously to the tumor binding domain (such as BCMA) of MM cells and CD3-positive T cells, resulting in perforin and granzymes-mediated MM cell lysis [29]. Even though multiple bispecific antibodies are being investigated in different clinical trials, only teclistamab got FDA approval for use in adult RRMM patients, who have received at least four lines of therapy, including a PI, IMiD, and anti-CD38 monocloncal antibody [13]. The approval came after the MajesTEC-1 trial (NCT03145181 and NCT04557098), which evaluated 165 RRMM patients with a median age of 64. Patients had a median of five prior LOT, with 78% of the included population being triple refractory. A total of 43% of the patients achieved ≥ CR after an mFU of 22 months. The mDOR, mPFS, and mOS were 24, 12.5, and 21.9 months, respectively [17]. Moreover, another BsAb named elranatamab received FDA breakthrough therapy designation based on the result of a multicentered phase II MagnetisMM-3 study (NCT04649359) [30]. In this study, 123 patients were enrolled; 96.7% were triple-refractory and 42.3% were penta-refractory. The ORR was recorded at 61%, with CR/sCR seen in 31.7%, VGPR in 23.6%, and PR in 5.7% of patients after an mFU of 12.8 months [31].

Antibody–drug conjugates (ADCs) are monoclonal antibodies (mAbs) bound to a toxin through a protein-based linker, causing targeted death of malignant MM cells and mildly affecting the healthy cells [14]. Belantamab mafodotin (blenrep) (GSK2857916) is an ADC investigated in the DREAMM-2 trial (NCT03525678) on heavily treated RRMM patients. In the 2.5 mg/kg group (*n* = 97), the ORR was recorded at 32%, with 19% achieving ≥ VGPR. The mDOR, mPFS, and mOS were recorded at 12.5, 2.8, and 15.3 months, respectively. For the cohort who received blenrep at 3.4 mg/kg (*n* = 99), the ORR was recorded at 35%, with 24% of patients achieving ≥ VGPR. The mDOR, mPFS, and mOS were recorded at 6.2, 3.9, and 14 months, respectively [18]. Based on this study, the FDA approved blenrep for RRMM patients in August 2020 [32]. DREAMM-14 (NCT05064358) is currently investigating whether alternative dosing and/or schedule regimens improve overall benefit or risk profile [33]. In November 2022, the DREAMM-3 (NCT04162210) trial did not meet its primary endpoint of PFS, upon which it is being withdrawn from the US market for RRMM patients [12]. DREAMM-3 is an open-label randomized phase III study (*n* = 325) that compared blenrep against pomalidomide and low-dose dexamethasone (Pd) in RRMM cases. The mPFS was longer for blenrep at 11.2 months compared to 7 months for Pd, which resulted in its withdrawal [34]. However, there are many phase I/II/III clinical trials are ongoing on blenrep either as monotherapy or combination therapy in RRMM such as, DREAMM 4 (NCT03848845), DREAMM 5 (NCT04126200), DREAMM 6 (NCT03544281), DREAMM 7 (NCT04246047), DREAMM 8 (NCT04484623), DREAMM 9 (NCT04091126), DREAMM 12 (NCT04398745), and DREAMM 13 (NCT04398680), which may provide another avenue of approval [35].

## 4. Complications Post-BCMA Therapy and Treatment

Even though BCMA-directed therapies have shown excellent efficacy in RRMM, the side effect profile is unique and requires management expertise. In this review, we will focus on the most common side effects of BCMA therapy and discuss their treatment in detail (Table 1).

### 4.1. Cytopenia(s)

The pathogenesis of cytopenia post-BCMA is unknown; however, the potentially implicated factors are multiple prior lines of therapy/lymphodepleting therapy, baseline cytopenia, hypogammaglobulinemia, myelodysplastic syndrome (MDS), high-grade CRS, high tumor burden at the time of treatment, high prevalence of clonal hematopoiesis of indeterminate potential (CHIP), and history of hemopoietic stem cell transplantation [37,38,39]. In the KarMMa-2 trial, the incidence of neutropenia was recorded at 80.6%, leukopenia in 29%, and anemia in 22.6% patients [15]. In the CARTITUDE-1 trial, neutropenia was observed in 95.9% of the patients, anemia in 81.4%, thrombocytopenia in 79.4%, leukopenia in 61.9%, and lymphopenia in 53.6% following cilta-cel administration [16]. In the MajesTEC-1 trial, 72% had neutropenia, 54% had anemia, 42% had thrombocytopenia, and 35% had lymphopenia [17]. Whereas, regarding blenrep, 21% developed anemia and 19% thrombocytopenia in the 2.5 mg/kg cohort, and 28% anemia and 29% thrombocytopenia in the 3.4 mg/kg cohort [18]. From real-world experience, anemia was seen in 100% of patients (88% G ≥ 3), neutropenia in 100% (100% G ≥ 3), thrombocytopenia in 94% (75% G ≥ 3), febrile neutropenia in 69% after ide-cel (*n* = 16) administration; anemia in 25.8% (10.9% G ≥ 3), thrombocytopenia in 23% (17.7% G ≥ 3), neutropenia in 15.7% (13.3% G ≥ 3) after cilta-cel; neutropenic fever in 6% following teclistamab (*n* = 17); thrombocytopenia in 27.4% (17.9% G ≥ 3), anemia in 11.3% (3.8% G ≥ 3), and neutropenia in 7.5% (4.7% G ≥ 3) following blenrep (*n* = 106) therapy [40,41,42,43].

Prophylaxis against cytopenia(s) [44]:

- Daily complete blood count (CBC) with differentials, basic metabolic panel, coagulation profile, ferritin is performed.

Management of cytopenia(s) [45,46,47,48,49,50]:

- In most cases, cytopenia(s) is self-limiting and needs watchful observation.
- For the symptomatic or severe cases of cytopenia(s), supportive management with blood product transfusion (packed red blood cell transfusion for Hgb < 7 and platelet transfusion if count < 10 K) is the mainstay. Usually, leuko-reduced and irradiated blood products are used for patients receiving CAR-T therapy.
- For thrombocytopenia refractory to transfusion, romiplostim or eltrombopag could be used.
- The use of granulocyte colony-stimulating factor (G-CSF) during periods of neutropenia is controversial. Some studies support using it to shorten periods of neutropenia decreasing the risk of infection; in contrast, others recommend against it as it increases the risk of CRS.
- It is recommended to perform a bone marrow biopsy in refractory or prolonged cytopenia(s) to rule out MDS, primary malignancy, AML, or persistence of myeloma.
- Autologous stem cell infusion has promising effects in refractory or prolonged cytopenic cases.

### 4.2. Infection

Cytopenia and infection are common complications post-BCMA therapy and are strongly co-related; however, infection can still happen without cytopenia. As per the population-based study conducted on 9253 MM patients over a period from 1988 to 2004, it was found that there was a seven-fold increased risk of any infections in MM patients compared to the general population [51]. In patients receiving BCMA therapy, the risk of infection further increases, and the factors responsible are the same as cytopenia [52]. The pathogenesis involved are immune exhaustion, decreased bone marrow reserve before receiving anti-BCMA therapy, and delay in immune recovery and neutropenia post-therapy [53]. The incidence of overall infection was recorded at 78%, with respiratory infections at 56%, COVID-19 in 27%, other viral in 10%, fungal in 5%, pneumocystis in 4%, and hepatitis B in 0.6% of patients [17]. From real-world experience, infection was seen in 31%, 28.6%, 35%, and 1.9% of patients following ide-cel, cilta-cel, teclistamab, and blenrep administration, respectively [40,41,42,43].

Prophylaxis against infection [53,54,55,56,57]:

- Antiviral prophylaxis with acyclovir 400 mg or valacyclovir 500 mg two times a day started on day 0 of CAR-T/BsAb and continued until absolute CD4 > 200 cells/μL.
- Anti-bacterial prophylaxis with ciprofloxacin 500 mg orally twice a day (or equivalent for quinolone-intolerant patients) initiated on day 0 of CAR-T and continued until ANC > 1000/μL. Regarding BsAbs, it is recommended to start prophylaxis at the onset of therapy, continue for the first month, and anytime ANC < 500/μL.
- Antifungal prophylaxis, such as fluconazole 200 mg daily or micafungin 50 mg IV daily in fluconazole-contraindicated cases, to be given during periods of prolonged and recurrent neutropenia.
- Pneumocystis prophylaxis with trimethoprim 160 mg-sulfamethoxazole 800 mg (double strength) orally three times per week given on day seven post-therapy of CAR-T/BsAb and continued until CD4 count is >200 cells/µL. Alternatives include dapsone 100 mg PO daily, pentamidine 300 mg inhaled monthly, or atovaquone 1500 mg PO daily.
- Baseline serologic testing or polymerase chain reaction (PCR) for hepatitis B/C virus (HBV/HCV), Epstein–Barr virus (EBV), cytomegalovirus (CMV), and human immunodeficiency virus (HIV).
- Patients who are carriers of HBV or have a history of prior infection should receive entecavir 0.5 mg PO daily until six months post CAR-T along with a periodic liver function or HBV DNA testing.
- The use of prophylactic IVIG during the period of hypogammaglobulinemia is controversial. After considering risk vs. benefit, the suggestion is to replace with intravenous (IV) IVIG 400–500 mg/kg if serum IgG ≤ 400 mg/dL. IVIG can also be considered in patients with serum IgG 400–600 mg/dL only during severe or recurrent infections.
- Immunization with killed/inactivated vaccines ≥ 6 months post CAR-T, or ≥2 months post last IVIG. Regarding live vaccine, it is recommended to give ≥ 1-year post-CAR-T therapy. Patients are also recommended to receive influenza and COVID-19 vaccinations; they can take repeated doses 3–6 months post CAR-T therapy. Pneumococcal vaccination must be administered before BsAbs, and 3–6 months post-CAR-T.
- In some centers, tixagevimab/cilgavimab is being given as a pre-exposure prophylaxis for SARS-CoV-2 to prevent symptomatic infections.

Management of infection [54,57]:

- Empiric broad-spectrum antibiotics to treat infection. CBC, chest X-ray, blood culture, computed tomography (CT) scan of chest/abdomen, urine analysis, and culture to de-escalate antibiotics.
- Infectious disease consultation.
- PCR CMV testing for patients with unexplained fever, cytopenia, pneumonitis, hepatitis, and colitis.

### 4.3. Cytokine Release Syndrome (CRS)

CRS is a supraphysiologic inflammatory response of the immune system. Although little is known about the pathophysiology of CRS in the case of bispecific antibody or CAR-T, it is considered to be due to the binding of these agents to their target resulting in the secretion of interferon-gamma (IFN-γ), tumor-necrosis-factor-alpha (TNF-α), granulocyte-monocyte colony-stimulating factor (GM-CSF), catecholamines, which activates both immune (macrophage) and non-immune (endothelial) cells [58,59]. Activated macrophages and endothelial cells produce many cytokines, such as IL-6, IL-1, nitric oxide (NO), and TNF-α, but IL-6 is pivotal in CRS [58,59,60,61]. The degree of elevation of IL-6 correlates with the severity of CRS [60]. These cytokines further activate endothelial cells, leading to capillary leakage and resistant hypotension, activate complement pathways and coagulation cascade, and inhibit myocardial function leading to cardiomyopathy [58,62,63,64]. 

The risk factors associated with severe cases are high disease burden, lymphodepletion with fludarabine and cyclophosphamide conditioning regimen, high CAR-T cell doses, thrombocytopenia before treatment initiation, and bulk CD8+ T cell selection without central memory [65]. The incidence of CRS was found to be 58.1% following ide-cel, 94.8% following cilta-cel, and 72% following teclistamab administration [15,16,17]. No incidence of CRS was recorded after blenrep administration [18]. From real-life experience, CRS was seen in 94% and 53% of patients following ide-cel and teclistamab administration, respectively [40,42].

CRS usually occurs 1–14 days after CAR-T therapy and 1–6 days after teclistamab administration [66,67]. The symptoms can range anywhere from mild self-limiting illnesses such as fever, myalgia, arthralgia, and anorexia to hemodynamic instability requiring vasopressors and mechanical ventilation [68,69]. Several scales, such as the Lee Criteria, the CARTOX Criteria, MSKCC criteria, CTCAE v4.03, CTCAE v5.0, ASTCT criteria, and the Penn Scale, are used to grade CRS based on patient’s signs and symptoms (Table 2) [44,68,70,71,72,73,74]. Due to overlapping symptoms, it is crucial to distinguish CRS from infection/sepsis and adrenal insufficiency [69].

Prophylaxis against CRS [19,20,21,44,46]:

- Patients can be given acetaminophen 650 mg and diphenhydramine 25–50 mg per oral (PO) before ide-cel and cilta-cel administration. In addition, patients are premedicated with dexamethasone 16 mg or equivalent before each dose of teclistamab to decrease the risk of CRS.
- Teclistamab is used in step-up doses, i.e., 0.06 mg/kg on day 1, 0.3 mg/kg on day 4, and 1.5 mg/kg on day 7 to lessen the risk of CRS.
- There is no role of prophylactic use of G-CSF on CRS.

Laboratory monitoring with CBC, basic metabolic panel, renal and hepatic function tests, coagulation profile, erythrocyte sedimentation rate (ESR), C-reactive protein (CRP), ferritin, and cytokine levels.

Management of CRS [19,20,21,44]:

- The management of CRS is mentioned in Table 3.

### 4.4. Immune Effector Cell Associated Neurotoxicity Syndrome (ICANS)

ICANS can range from mild headache, confusion, and somnolence to life-threatening situations, including seizures, cerebral edema, and comatose condition [66]. The risk factors associated with ICANS are young age, increased disease burden, severe CRS, high dose of CAR-T cells, and pre-existing neurologic disorder [75]. Similarly to CRS, the pathogenesis of ICANS involves cytokines such as IL-1, IL-2, IL-3, IL-5, IL-6, IL-10, GM-CSF, and IFN-γ; the higher peak concentration of these cytokines is associated with severity of neurotoxicity [76]. Moreover, a xenograft mouse model showed the resolution of ICANS following IL-1 blockage by anakinra but not through IL-6 blockage, demonstrating monocyte activation through both IL-1 and IL-6, causing systematic inflammation [77]. Another mechanism involved in ICANS is the disruption of the blood–brain barrier (BBB), evident by high levels of protein and leukocyte count in cerebrospinal fluid (CSF) [75]. Moreover, there were increased levels of cytokines such as IL-6, IFN-γ, TNF-α, and GM-CSF in the cerebrospinal fluid, indicating either the disruption of the BBB or increased production in CSF [75]. These cytokines are responsible for astrocyte injury, increasing astrocyte markers such as glial fibrillary acidic protein (GFAP) and calcium-binding protein B (S100B) in the CSF following CAR-T [75]. Finally, endothelial activation evident by an increase in angiotensin 2: angiotensin 1 ratio in response to inflammatory cytokines also contributes to ICANS by causing further disruption of BBB, vascular dysfunction, increased capillary permeability, and coagulation cascade activation [75]. The incidence of ICANS is recorded as 6.5% following ide-cel, 21.6% following cilta-cel, and 3% following teclistamab administration [15,17,36]. No incidence of ICANS was recorded after blenrep administration [18]. From real-world experience, ICANS was seen in 6%, 19.8%, and 6% of patients following ide-cel, cilta-cel, and teclistamab administration, respectively [40,41,42].

The onset of ICANS is typically within five days of CAR-T and 2–8 days of teclistamab administration [44,67]. CTCAE v5.0 and CRES are the scales commonly used to grade ICANS (Table 4) [44,71].

Prophylaxis against ICANS [44]:

- Levetiracetam 750 mg PO two times a day (or equivalent in intolerant patients) started on day 0 and continued until day 30 for seizure prophylaxis post-CAR-T and BsAbs.

Management of ICANS [19,20,21,44]:

- The management of ICANS post-BCMA therapy is mentioned in Table 5.

### 4.5. Hemophagocytic lymphohistiocytosis/Macrophage activation Syndrome (HLH/MAS)

HLH/MAS is a life-threatening immunological disorder that could happen due to primary (familial) or secondary (malignancy, infection, autoimmune disorder) etiologies [78]. These triggers result in the hyperactivation of cytotoxic T lymphocytes (CTL) and natural killer (NK) cells, causing them to over-activate and hyper-proliferate the antigen-presenting cells such as macrophages [78]. Macrophages secrete cytokines such as IFN-γ, IL-1, IL-2, IL-6, TNF-α, which send further positive feedback to CTLs and NK cells [78,79]. This hypersecretion of cytokines is termed as a cytokine storm, which could contribute to tissue damage and systemic organ failure, leading to the development of clinical and laboratory features of HLH [80]. HLH/MAS represents a complication of CAR-T therapy among anti-BCMA therapy (carHLH), the exact pathophysiology of which is yet to be found in the literature. However, Lichtenstein et al. found pathologic T cell expansion, high IFN-γ, IL-1, IL-6, IL-8, IL-10, IL-18 levels in peripheral blood (PB) and bone marrow (BM) of 59 patients with carHLH [81]. It is imperative to differentiate HLH/MAS from sepsis, ICANS, or CRS, given the overlapping signs and symptoms [44]. To diagnose carHLH/MAS (which is different from primary or secondary HLH/MAS), patients must have ferritin > 10,000 ng/mL, with any two of the following organ toxicities: grade ≥ 3 increase in liver enzymes or serum bilirubin, grade ≥ 3 increase in serum creatinine, grade ≥ 3 pulmonary edema, presence of hemophagocytosis in the bone marrow or other organs [44]. The incidence of carHLH/MAS was found to be 1% following CAR-T administration [44].

Management of carHLH/MAS [44,82,83,84]:

- IV tocilizumab and IV steroid according to the grade 3 CRS algorithm (Table 1).
- Assess improvement by checking lactate dehydrogenase (LDH) levels, blood ferritin level, fibrinogen, liver enzymes, and kidney function. If there is no improvement of the above parameters in 48 h, one can either add etoposide 75–100 mg/m^2^ (can be repeated every 4–7 days depending on the clinical response) or intrathecal cytarabine along with hydrocortisone for carHLH-related neurotoxicity.
- Anakinra, a recombinant IL-1 receptor antagonist, is also recommended, given its short half-life and favorable side effects profile.
- Ruxolitinib, a Janus kinase 1/2 inhibitor, is also being used in some small studies, as blockage of the pathway can suppress the inflammatory cytokines.

### 4.6. Keratopathy

Keratopathy is described as changes in the corneal epithelium with or without visual changes. It is hypothesized that blenrep initially enters the cornea through the vessels in the limbus or the tear film and then enters corneal epithelial cells through micropinocytosis [85,86]. This eventually results in apoptosis and the extrusion of corneal epithelial cells [85,86]. In the DREAMM-2 trial, keratopathy was the most common complication, occurring in 71% of the cases [18]. In this study, a change in visual acuity was seen in 21%, blurred vision in 23%, and dry eye in 15% of the cases [18]. From real-world experience, keratopathy was seen in 68.4% of patients (40% G ≥ 3) and blurred vision in 36.8% (6.3% G ≥ 3) following blenrep administration [43].

Prophylaxis/monitoring against keratopathy [22,86]:

- All patients should have a baseline eye exam, which includes a visual acuity assessment and slit lamp examination. The findings are combined and graded based on keratopathy and visual acuity scale (KVA). The exam should be repeated before every use and with any changes in vision while taking blenrep.

Management of keratopathy [22,86]

- To continue blenrep for grade 1 or mild superficial keratopathy (mild loss in visual acuity, one-line reduction in Snellen chart from baseline).
- For grade 2 or moderate superficial keratopathy (2–3-line reduction from baseline not worse than 20/200), one should withhold blenrep until the patient reaches grade ≤ 1 and then resume at a similar dose.
- For grade 3 or severe superficial keratopathy (>3 lines reduction from baseline not worse than 20/200), one should withhold blenrep until grade ≤ 1 and then resume at a lower dose.
- For grade 4 keratopathy (corneal erosion/ulceration, visual acuity < 20/200), one can either follow the recommendation for grade 3 keratopathy or discontinue medication indefinitely.

### 4.7. Other Complications of Ciltacabtagene

Among other adverse effects commonly seen are fatigue (37.1%), cough (35.1%), hypocalcemia (32%), hypophosphatemia (30.9%), diarrhea (29.9%), decreased appetite (28.9%), nausea (27.8%), hypoalbuminemia (27.8%), increased liver enzymes (24.7–28.9%), constipation (22.7%), hyponatremia (22.7%), hypokalemia (20.6%), pyrexia/chills (20.6%) [36]. The management of these complications is not mentioned in this trial; however, these could be manifestations of the complications discussed above or present as isolated symptoms needing supportive management.

## 5. Conclusions

MM is the second most common hematologic malignancy, which remains incurable despite advances in its treatment secondary to drug resistance and the presence of minimal residual disease. Recently, BCMA agents have shown favorable outcomes in heavily pre-treated RRMM cohorts, as evidenced by multiple published clinical trials. Despite being targeted immunotherapies, these are not free from adverse effects. With the increasing age, multiple prior lines of therapy, and baseline ECOG performance status, some patients might be more susceptible to the adverse effects than others. Constant monitoring, early identification of worsening symptoms, and prompt response might mitigate the complication with favorable outcomes. However, further phase III clinical trials with longer follow-up periods are needed to establish the safety of such novel agents in this vulnerable population.

## Figures and Tables

**Table 1:** Most common complications post-BCMA [15,17,18,36].

Trial Name, ID	Agent	Main Side Effects, %	Grade ≥ 3 (%)
KarMMa-2, NCT03361748 (Dhodapkar M, 2022, Blood) [15]	Idecabtagene Vicleucel (ide-cel)	Neutropenia, 80.6% Leukopenia, 29% Anemia, 22.6% CRS, 58.1% ICANS, 6.5%	80.6% 29% 22.6% 0 3.2%
CARTITUDE-1, NCT03548207 (Martin T, 2022, ASCO) [36]	Ciltacabtagene autoleucel (cilta-cel)	Neutropenia, 95.9% Anemia, 81.4% Thrombocytopenia, 79.4% Leukopenia, 61.9% Lymphopenia, 53.6% CRS, 94.8%	94.8% 68% 59.8% 60.8% 50.5% 4.1%
MajesTEC-1, NCT03145181, NCT04557098 (Moreau P, 2023, ASCO) [17]	Teclistamab	Neutropenia, 72% Anemia, 54% Thrombocytopenia, 42% Lymphopenia, 35% Infection, 78% CRS, 72% ICANS, 3%	65% 38% 22% 33% 52% 0.6% 0
DREAMM-2, NCT03525678 (Nooka AK, 2022, Blood) [18]	Belantamab (2.5 mg/kg) (blenrep)	Keratopathy, 71% Blurred vision, 23% Reduced visual acuity, 21% Dry eye, 15% Anemia, NR Thrombocytopenia, NR	29% NR NR NR 21% 19%

NR—not recorded.

**Table 2:** Grading systems of CRS [44,68,70,71,72,73,74].

Grades	Lee Criteria	CARTOX Criteria	MSKCC Criteria	CTACAE v4.03	CTCAE v5.0	ASTCT Criteria	Penn Scale
Grade 1	Fever, headache, nausea, malaise	Fever, grade 1 organ toxicity	On antipyretics, antiemetics, analgesics	Can continue infusion as mild reaction	Fever	Temperature ≥ 38 °C, No hypotension/hypoxia	Supportive care (antipyretics/antiemetics) due to mild reaction
Grade 2	FiO_2_ < 40%, requiring fluids/ low dose of one vasopressor, grade 2 organ toxicity	FiO_2_ < 40%, on fluids/low dose vasopressor, grade 2 organ toxicity	FiO_2_ < 40%, on vasopressor < 24 h.	Infusion interruption, symptomatic treatment with analgesics/antipyretics/antiemetics, prophylactic medication if ≤24 h of onset.	FiO_2_ < 40%, hypotensive on fluids.	Temperature ≥ 38 °C, hypotension not requiring vasopressors, hypoxia requiring low-flow oxygen	Hospitalization needed for moderate reaction, need IV therapies/parenteral nutrition, grade 2 organ toxicity or grade 3 transaminitis
Grade 3	FiO_2_ ≥ 40%, on high dose/multiple vasopressors, grade 3 organ toxicity or grade 4 transaminitis	FiO_2_ ≥ 40%, on high dose/multiple vasopressors, grade 3 organ toxicity or grade 4 transaminitis	FiO_2_ ≥ 40%, on vasopressors ≥ 24 h	Infusion interruption, hospitalization	FiO_2_ ≥ 40%, on one vasopressor	Temperature ≥ 38 °C with requiring vasopressor, and/or requiring high-flow oxygen	Hospitalization for more severe reaction, grade 3 organ toxicity or grade 4 transaminitis, hypoxia requiring O_2_, hypotension requiring IV fluid or low dose pressors, coagulopathy requiring FFP or cryoprecipitate.
Grade 4	On MV, grade 4 organ toxicity	On MV, grade 4 organ toxicity	On MV, hypotensive despite high-dose vasopressors	MV, pressor support needed	Urgent intervention needed due to life-threatening condition	Temperature ≥ 38 °C, requiring multiple vasopressors, on PPV (CPAP, BiPAP, MV)	On MV, hypotensive requiring high dose vasopressors

MV—mechanical ventilator, O_2_—oxygen, PPV—positive pressure ventilation, CPAP—continuous positive airway pressure, BiPAP—Bilevel positive airway pressure, IV—intravenous.

**Table 3:** The management of CRS post CAR-T and BsAbs [19,20,21,44].

Grade	Treatment of CRS
Grade 1	Supportive management such as, acetaminophen/NSAIDs for fever, intravenous fluid (IVF) for hydration, empiric antibiotics for suspected/confirmed infection. Tocilizumab 8 mg/kg intravenous (IV) over 1 h (maximum 800 mg) May consider dexamethasone 10 mg IV daily. For teclistamab: Discontinue agents and administer premedication before subsequent doses
Grade 2	Supportive management such as oxygen supplementation to maintain saturation ≥ 92%, 2–3 L IVF boluses to target systolic blood pressure > 90 mm Hg Tocilizumab 8 mg/kg IV every 6–8 h (maximum three doses/day) for refractory hypotension, hypoxemia, or organ toxicity. For unresponsive cases, transfer to the intensive care unit (ICU) for vasopressor support, check inferior vena cava collapsibility index, echocardiogram, and place arterial line to monitor mean arterial pressure (MAP). Dexamethasone 10 mg IV every 12–24 h. For refractory cases, dexamethasone 20 mg IV every 6–12 h, or methyl prednisone 2 mg/kg every 12 h. Management of organ toxicity as per institutional guidelines.
Grade 3	Patients may need high-flow oxygen or non-invasive positive pressure ventilation (NIPPV) for hypoxemia. For hypotension, 1–2 vasopressors, also dexamethasone 10 mg IV every 6 h. Can be increased to 20 mg IV every 6 h in unresponsive cases. For teclistamab: Discontinue temporarily if first episode, and permanently if recurrent episodes
Grade 4	Mechanical ventilation, continuous veno-venous hemodialysis for hypoxemia refractory to high-flow or NIPPV. Multiple vasopressors for refractory hypotension to keep MAP > 65. Methyl prednisone 1–2 g IV daily For teclistamab: Discontinue indefinitely

**Table 4:** Grading systems of ICANS [44,71].

Grades	CTCAE v 5.0	CARTOX Criteria
Grade 1	Encephalopathy, tremor, headache, confusion, depressed consciousness: Mild symptoms Seizure: Brief partial seizure with intact consciousness Dysphasia: aware of receptive/expressive characteristics, intact communication.	Neurologic assessment score: Mild impairment, scoring 7–9
Grade 2	Encephalopathy, tremor, headache, confusion, depressed consciousness: moderate symptoms/disorientation, limiting instrumental activities of daily living (ADL), slow response to stimuli, sedation. Seizure: Brief generalized seizure. Dysphasia: moderate receptive/expressive characteristics, impaired spontaneous communication	Neurologic assessment score: Moderate impairment, scoring 3–6
Grade 3	Encephalopathy, tremor, headache, confusion, depressed consciousness: severe symptoms/disorientation, difficult to arouse, limiting ADL. Seizure: New onset multiple seizures despite treatment. Dysphasia: Severe receptive/expressive characteristics, impaired communication/reading/writing Cerebral edema: new onset, worsening from baseline	Neurologic assessment score: Severe impairment, scoring 0–2. Increased intracranial pressure (ICP): Stage 1–2 papilledema or CSF opening pressure < 20 mmHg. Seizure: Partial or non-convulsive seizures responding to benzodiazepine
Grade 4	Life-threatening condition needing urgent neuro-intervention	Neurologic assessment score: Obtunded Increased ICP: Stage 3–5 papilledema or CSF opening pressure ≥ 20 mm Hg, or cerebral edema Seizure: Generalized seizure or status epilepticus, new weakness

**Table 5:** The management of ICANS post CAR-T and BsAbs [19,20,21,44].

Grade	Treatment
Grade 1	Supportive measures such as IV fluid and medication, aspiration precaution, nothing by mouth, parenteral nutrition. Judicious use of lorazepam 0.25–0.5 mg IV every 8 h or haloperidol 0.5 mg IV every 6 h only for agitated patients. Workup including fundoscopy to look for papilledema, CT head or MRI brain for cerebral edema, and electroencephalogram (EEG) to assess seizure. Dexamethasone 10 mg IV once or twice daily Tocilizumab 8 mg/kg IV if concurrently with CRS. For teclistamab: Discontinue agents and administer premedication before subsequent doses
Grade 2	As per grade 1 ICANS, transfer to ICU. Dexamethasone 10 mg IV every 12 h for 48–72 h. For unresponsive cases, can increase dexamethasone to a maximum of 20 mg IV every 6 h.
Grade 3	As per grade 1 ICANS. In the ICU, dexamethasone 10–20 mg IV every 6 h. For unresponsive cases, can give methyl prednisone 1–2 g/day. For stage 1 or 2 papilloedema without cerebral edema: bolus dose of acetazolamide 1000 mg IV, followed by maintenance dose of 250–1000 mg IV two times a day. Repeat CT head or MRI brain in patients with persistent grade ≥ 3 ICANS. For teclistamab: Discontinue temporarily if first episode, and permanently if recurrent episodes
Grade 4	As grade 3. For stage ≥ 3 papilloedema, CSF pressure ≥ 20 mmHg, focal or diffused cerebral edema: Methylprednisone IV 1 g/day, elevate head end to 30 degrees, hyperventilation to target PaCO_2_ of 28–30 mmHg. Mannitol 0.5–1 g/kg with a maintenance of 0.25–1 g/kg every 6 h, or 250 mL 3% hypertonic saline with maintenance at 50–75 mL/h. One needs to serially monitor serum osmolality and electrolytes for mannitol and hypertonic saline, respectively. Can give 30 mL of 23.4% hypertonic saline for imminent herniation. Drain CSF to reduce pressure < 20 mmHg. For status epilepticus: For bolus: Lorazepam 0.5–2 mg IV every 5 min, with a total of 2–4 mg, and levetiracetam 500 mg IV. For refractory cases: can load with phenobarbital 60 mg IV for the non-convulsive subtype, and 15 mg/kg IV for the convulsive subtype. For maintenance: lorazepam 0.5 mg IV every 8 h, or levetiracetam 1000 mg IV every 12 h, or phenobarbital 30 mg IV every 12 h for the non-convulsive type or 1–3 mg/kg IV every 12 h for the convulsive type. For teclistamab: Discontinue indefinitely. Neurology or neurosurgery consultation.

## Data Availability

No new data were created or analyzed in this study. Data sharing is not applicable to this article.
